# Psychische und neurokognitive Erkrankungen und Diabetes mellitus (Update 2026)

**DOI:** 10.1007/s00508-025-02654-z

**Published:** 2026-04-30

**Authors:** Heidemarie Abrahamian, Alexandra Kautzky-Willer, Angelika Rießland-Seifert, Alexander Kautzky, Johanna Brix, Birgit Harb, Dora Beer, Diana Lebherz-Eichinger, Peter Fasching, Hermann Toplak

**Affiliations:** 1 Endokrinologische Ordination, Wien, Österreich; 2 Gender Medicine-Unit, Klinische Abteilung für Endokrinologie und Stoffwechsel, Universitätsklinik für Innere Medizin III, Medizinische Universität Wien, Wien, Österreich; 3 Ordination für Psychiatrie und Psychotherapie, Wien, Österreich; 4 Klinische Abteilung für Sozialpsychiatrie, Universitätsklinik für Psychiatrie und Psychotherapie, Medizinische Universität Wien, Wien, Österreich; 5 Division of Insurance Medicine, Department of Clinical Neuroscience, Karolinska Institute, Stockholm, Schweden; 6 1. Medizinische Abteilung mit Diabetologie, Endokrinologie und Nephrologie, Klinik Landstraße, Wiener Gesundheitsverbund, Wien, Österreich; 7 Reha-Zentrum St. Radegund, St. Radegund, Österreich; 8 Freie Praxis, Graz, Österreich; 9 Diabeteszentrum Wienerberg, Wien, Österreich; 10 4. Medizinische Abteilung – Innere Medizin mit Kardiologie, Klinik Hietzing, Wiener Gesundheitsverbund, Wien, Österreich; 11 5. Medizinische Abteilung mit Endokrinologie, Rheumatologie und Akutgeriatrie mit Ambulanz, Klinik Ottakring, Wiener Gesundheitsverbund, Wien, Österreich; 12 Endokrinologie und Diabetologie, Graz, Österreich

**Keywords:** Diabetes mellitus, Diabetes distress, Demenz, Essstörungen, Depression, Diabetes mellitus, Diabetes distress, Dementia, Eating disorders, Depression

## Abstract

Diabetes mellitus ist häufig mit psychischen Erkrankungen assoziiert. Depressive Störungen kommen bei Patient:innen mit Diabetes doppelt so häufig vor wie in der nichtdiabetischen Population. Andere psychische Erkrankungen, die gehäuft mit Prädiabetes und Diabetes mellitus auftreten, sind kognitive Dysfunktionen bis zur Demenz, auffälliges Essverhalten, Angststörungen, Schizophrenie, bipolare Störungen, Aufmerksamkeitsdefizit/Hyperaktivitätsstörung (ADHS) und emotional instabile Persönlichkeitsstörungen. Die ungünstigen Auswirkungen dieser Komorbiditäten auf den Stoffwechsel sind nachhaltig und manifestieren als schlechtere metabolische Kontrolle und vermehrte mikro- und makroangiopathische Komplikationen. Ziel dieses Positionspapieres ist die Sensibilisierung aller involvierten medizinischen Fachkolleg:innen sowie aller anderen mit dem Thema Diabetes befassten Berufsgruppen und Organisationen, um eine Intensivierung der komplexen therapeutischen Interventionen bei betroffenen Patient:innen zu erreichen. Positive Auswirkungen wären zum einen eine geringere Inzidenz von Diabetes mellitus bei Patient:innen mit psychischen Erkrankungen und zum anderen eine Reduktion diabetesspezifischer Folgeerkrankungen – insbesondere der kardiovaskulären Morbidität und Mortalität – sowie eine verbesserte Lebensqualität bei Menschen mit Diabetes und komorbider psychischer Erkrankung.

## Abstract (en)

Diabetes mellitus is frequently associated with mental diseases. Depressive disorders are twice as frequent in patients with diabetes compared to the nondiabetic population. Other mental diseases that frequently occur with diabetes and prediabetes are cognitive impairment up to dementia, disturbed eating behavior, anxiety disorders, schizophrenia, bipolar disorders, attention deficit/hyperactivity disorder (ADHD) and borderline personality disorder. The unfavorable effects of these comorbidities on metabolism are lasting and are manifested as poorer metabolic control and increased microangiopathic and macroangiopathic complications. The aim of this position paper is to raise awareness of all medical specialists as well as all other professional groups and organizations involved in the topic of diabetes to achieve an intensification of the complex treatment interventions in affected patients. Positive effects would include a reduced incidence of diabetes mellitus in patients with mental disorders and a reduction of diabetes-specific complications, particularly cardiovascular morbidity and mortality, as well as an improved quality of life in individuals with diabetes and comorbid mental illness.

## 1.1 Einleitung

Zwischen Diabetes mellitus und bestimmten psychischen Erkrankungen besteht eine bidirektionale Beziehung. Depressionen, Angststörungen, Essstörungen und neurokognitive Defizite treten in der diabetischen Population gehäuft auf [1]. Die Zusammenhänge sind teilweise bekannt. Darüber hinaus können bestimmte psychische Erkrankungen wie Schizophrenie, schizotype und wahnhafte Störungen sowie bipolare Störungen zu einer erhöhten Inzidenz von Diabetes mellitus führen. Die Ursache dürfte in charakteristischen Besonderheiten der psychiatrischen Erkrankung und in potenziellen Nebenwirkungen von bestimmten Psychopharmaka begründet sein [1–4].

Menschen mit somatischer Morbidität in Kombination mit einer schweren psychischen Erkrankung weisen eine signifikant kürzere Lebenserwartung und eine zwei- bis dreifach erhöhte Mortalitätsrate im Vergleich zu psychisch gesunden, nur somatisch erkrankten Patient:innen auf [5]. Die Häufigkeit des Auftretens von psychischen Komorbiditäten ist bei Typ-1-Diabetes und Typ-2-Diabetes unterschiedlich und hängt unter anderem auch mit den jeweiligen pathophysiologischen und psychopathologischen Hintergründen zusammen [5].

In einer groß angelegten Studie (Treatment Options for Type 2 Diabetes in Adolescents and Youth [TODAY2] study) wurde die Prävalenz von Depression, Essstörungen und verringerter gesundheitsbezogener Lebensqualität („health related quality of life“ [HRQOL]) bei jungen Erwachsenen mit Manifestation von Typ-2-Diabetes in der Jugend erhoben [6]. Die Symptome von Depression und beeinträchtigter HRQOL waren initial häufig und nahmen über die Beobachtungsdauer signifikant zu (Depression von 14,0 % auf 19,2 %, *p* < 0,003; beeinträchtigte HRQOL von 13,1 % auf 16,7 %, *p* < 0,009). Depression und reduzierte HRQOL traten vermehrt bei Frauen und bei Patient:innen mit niedrigerem Familieneinkommen auf. Die Häufigkeit von Binge-Eating-Störungen blieb über den Beobachtungszeitraum konstant, Fälle von selbst berichtetem Insulin-Purging häuften sich. Symptome der Depression waren mit höheren HbA_1c_(glykiertes Hämoglobin)-Werten, mehr Hypertonie und ebenso mit einer Progression der Retinopathie assoziiert. Verringerte HRQOL wies eine Assoziation mit höherem BMI (Body Mass Index) , höherem systolischem Blutdruckwert und ebenso mit Progression der Retinopathie auf. Die TODAY2-Studie zeigt eindrucksvoll die Häufigkeit von psychischen Beeinträchtigungen bereits bei jungen Patient:innen mit in der Jugend manifestiertem Typ-2-Diabetes sowie die ungünstigen Auswirkungen auf den Verlauf des Diabetes mellitus [6].

Der Einfluss psychischer Erkrankungen auf die Qualität der Stoffwechselkontrolle bzw. auf kardiovaskuläre Risikofaktoren bei Diabetes mellitus ist in der Regel ungünstig und signifikant und beeinflusst die Entwicklung von mikro- und makrovaskulären Spätschäden [1, 5].

Ziel dieses Positionspapieres ist sowohl die Sensibilisierung aller involvierten medizinischen Fachkolleg:innen sowie sonstiger mit diesem Thema befasster Berufsgruppen und Organisationen als auch die Intensivierung der komplexen therapeutischen Interventionen bei Patient:innen mit Diabetes mellitus und komorbider psychischer Erkrankung. Analog den Anforderungen des publizierten Position-Statements der Amerikanischen Diabetes Gesellschaft sollte auch in Österreich eine umfassende psychosoziale Betreuung in die Diabetesbehandlung integriert werden und für alle Menschen mit Diabetes mellitus zugänglich gemacht werden [1].

Die Umsetzung dieses Positionspapieres soll dazu beitragen, einerseits die Inzidenz von Diabetes mellitus bei psychisch erkrankten Menschen zu senken und andererseits diabetesspezifische Folgeerkrankungen – insbesondere kardiovaskuläre – bei Menschen mit Diabetes und komorbider psychischer Erkrankung zu reduzieren und die Lebensqualität zu verbessern.

## 1.2 „Diabetes distress“

Die Anforderungen und Belastungen, die die Erkrankung Diabetes mellitus durch das erforderliche Selbstmanagement mit sich bringt, werden individuell unterschiedlich erlebt und bewertet und können zu einer erheblichen kognitiven und emotionalen Stressbelastung mit psychischer Beeinträchtigung führen. Das Auftreten von „diabetes distress“ („Diabetes-spezifischer Stress“) wird bei Typ-1- und Typ-2-Diabetes beschrieben. Psychische Symptome entwickeln vor allem Patient:innen, die durch das Selbstmanagement des Diabetes mellitus kognitiv und emotional überfordert sind. Häufig besteht ein Zusammenhang mit ungünstigen Lebensumständen [1]. Die diagnostischen Kriterien einer Depression werden dabei nicht erfüllt, obwohl teilweise überlappende Symptome bestehen. Da sich Diagnostik, Verlauf und Therapie wesentlich voneinander unterscheiden, ist eine klare Differenzierung der klinischen Bilder „Depression“ und „Diabetes-spezifischer Stress“ erforderlich [7].

Die Prävalenz von Diabetes-spezifischem Stress liegt mit 22–42 % aller an Typ-1- und mit über 60 % aller an Typ-2-Diabetes mellitus erkrankten Personen hoch [1, 2]. Da negative Auswirkungen auf HbA_1c_, Selbstwirksamkeit, Lebensqualität und Therapieadhärenz beschrieben werden, ist einerseits die Diagnostik wichtig, andererseits die zur Diagnose zeitnahe Teilnahme an einer Diabetesschulung [1, 6, 8]. In dieser Schulung soll der Umgang mit körperlichen, psychischen und sozialen Anforderungen, die mit der Erkrankung einhergehen, erlernt werden. Darüber hinaus ist zu prüfen, ob der Patient/die Patientin über ausreichende personelle, soziale und ökonomische Ressourcen verfügt und diese auch nutzen kann. Die Förderung von individueller Stressresilienz ermöglicht einen eigenverantwortlichen Umgang mit der Erkrankung. Für den/die Behandler:in stellt sich das Bild von diabetesbezogenem, nicht bewältigbarem Stress häufig auch als mangelnde Therapieadhärenz dar, wobei Überlastung und Überforderung der Patient:in dabei im Vordergrund stehen. Ist die Einschätzung bezüglich aktivierbarer Ressourcen und Stressresilienz negativ, sollte eine klinisch-psychologische und/oder psychotherapeutische Intervention als begleitende Maßnahme zur Seite gestellt werden.

Für die Diagnostik sind folgende Instrumente gut validiert und zu empfehlen: Problem Areas In Diabetes (PAID) umfasst 20 Fragen, die sowohl emotionalen Stress als auch spezifische Belastungen des Diabetes erfassen [1, 9]. Eine deutschsprachige Version steht zur Verfügung. Die Diabetes Distress Scale (DDS) umfasst 17 Fragen und erlaubt durch die Einbeziehung von 4 Bereichen wie emotionale Belastung, interpersonelle Belastung, Ärzt:in-bezogener Stress und Therapie-bezogener Stress eine umfassende Beurteilung mit einer soliden Bewertungsmöglichkeit [1, 10, 11].

## 1.3 Depression

Nach der ICD-10-Klassifikation (International Classification of Diseases, 10th Revision) unterscheidet man bei Patient:innen mit depressiven Symptomen leichte, mittelgradige und schwere depressive Episoden, weiters rezidivierende depressive Störungen, anhaltende affektive Störungen und andere seltenere affektive Störungen. Die ICD-10 wird in den kommenden Jahren schrittweise durch die ICD-11 abgelöst. In Österreich ist die Einführung derzeit in Planung und wird voraussichtlich zwischen 2025 und 2027 umgesetzt. Die wesentlichen Neuerungen bei ICD-11 sind unter anderen die Integration neuer Krankheitsbilder, die Ermöglichung einer flexibleren Diagnosestellung, die vollständige elektronische Nutzbarkeit sowie die stärkere Berücksichtigung kultureller und geschlechtsspezifischer Aspekte.

Die Beziehung zwischen Depression und Diabetes mellitus ist bidirektional und bildet damit eine Nahtstelle zwischen mehreren medizinischen Fachdisziplinen sowie Gesundheitsdienstleistern [1, 2, 12–16]. Eine Metaanalyse beschreibt das Risiko für das Erkranken an einer Depression für Patient:innen mit Typ-2-Diabetes mellitus mit 17,6 % als etwa doppelt so hoch im Vergleich zu einer nichtdiabetischen Kontrollgruppe mit 9,8 % [17]. Wie in der nichtdiabetischen Population sind Frauen häufiger betroffen als Männer (23,8 % versus 12,8 %). Daten der Gesundheitsversicherungen bestätigen diese Zahlen auch für Österreich, wobei auch hier für Frauen mit Diabetes eine deutlichere Risikoerhöhung für Depression (Odds Ratio [OR] 2,6) festgestellt wurde im Vergleich zu Männern mit Diabetes (OR 1,9) [18]. Ein exzellenter systematischer Review mit Metaanalyse beschreibt die Prävalenz der Depression für Menschen mit Typ-1-Diabetes mit 22 % (Vergleich ohne Diabetes 13 %) und für Menschen mit Typ-2-Diabetes mit 19 % (Vergleich ohne Diabetes 11 %). Diese Publikation bestätigt die Risikoerhöhung für Depression sowohl für Typ-1-Diabetes OR 2,10 als auch für Typ-2-Diabetes OR 1,76 [19].

Umgekehrt erhöht das Vorliegen einer Depression das Risiko, an Typ-2-Diabetes zu erkranken – das relative Risiko ist für Menschen mit Depression um 37–41 % höher als für Menschen ohne Depression [16, 20].

Diese wechselseitige bzw. bidirektionale Beziehung zwischen Diabetes mellitus und Depression wird durch komplexe pathophysiologische Mechanismen vermittelt. Auf biologischer Ebene spielen vor allem neuroendokrine und inflammatorische dysfunktionale Prozesse eine Rolle. Ursachen für die Induktion von subklinischer Inflammation sind beispielsweise viszerale Adipositas, reichliche Zufuhr von fett- und zuckerreichen stark verarbeiteten Nahrungsmitteln, chronischer psychosozialer Stress, Störungen des Darmmikrobioms und viele andere. Chronischer Stress führt zu einer Hyperaktivität der Hypothalamus-Hypophysen-Nebennieren-Achse und dem sympathischen Nervensystem mit konsekutiver Erhöhung von Cortisol und Katecholaminen, die zu einer Steigerung von Insulinresistenz und Blutzuckerwerten führen. Subklinische Inflammation geht mit erhöhter Produktion von bestimmten Zytokinen einher, die als wirksame Mediatoren die Blut-Hirn-Schranke passieren und direkt oder indirekt mit dem Neurotransmittersystem interferieren [21–23].

Proinflammatorische Prozesse durch vermehrte Zytokinproduktion im viszeralen Fettgewebe wurden auch für Adipositas, einer wesentlichen Vorerkrankung von Typ-2-Diabetes, nachgewiesen. Adipokine wie Leptin, Adipokin und Resistin werden im Fettgewebe produziert und sind in inflammatorische, metabolische und psychoendokrine Vorgänge involviert. Adipositas ist somit ein Risiko für Diabetes mellitus und für Depression [24].

Zusätzlich beeinträchtigt eine Hyperglykämie bei diabetischer Stoffwechsellage die regelrechte Funktion des Immunsystems und erhöht den oxidativen Stress, welcher neuroinflammatorische Prozesse weiter fördert und neurotoxisch wirkt. Darüber hinaus wurden mikrovaskuläre Prozesse bei der Depressionsentstehung infolge eines Diabetes mellitus nachgewiesen [21]. Neben diesen biologischen Mechanismen spielen psychosoziale Faktoren in der Beziehung zwischen Diabetes mellitus und Depression eine entscheidende Rolle. Vor allem sozioökonomisch benachteiligte Menschen haben durch die Einwirkung damit verbundener psychosozialer Stressfaktoren ein erhöhtes Risiko für metabolische wie auch psychische Erkrankungen. Geringe finanzielle Ressourcen, niedriges Bildungsniveau und mangelhafte soziale Einbettung machen Betroffene vulnerabel für verschiedene Stressoren, erschweren die Krankheitsakzeptanz und den Zugang zu Gesundheitsleistungen [2, 11, 25, 26].

Eine bestehende Depression kann die Krankheitsverarbeitung für Diabetes und die weitere Prognose erheblich beeinträchtigen. Oft können sich solche Patient:innen nicht an Ernährungsempfehlungen halten, machen wenig körperliche Bewegung und konsumieren häufiger Alkohol und Nikotin [11, 21, 25, 26].

Die publizierten Daten der Diabetes Attitudes Wishes and Needs Study 2 (DAWN2) bestätigen bei Patient:innen mit Diabetes mellitus individuell empfundene Belastungen bezogen auf Lebensqualität, Selbstmanagement, Einstellungen zur Krankheit sowie Defizite durch mangelhafte soziale Unterstützung über mehrere Länder hinaus [27]. Die Interferenz der Depression mit der Qualität der Stoffwechseleinstellung dürfte von der Anzahl und Schwere der psychischen Symptome linear abhängig sein [17, 27]. Daten der ACCORD (Action to Control Cardiovascular Risk in Diabetes)-Studie zeigen, dass bei diabetischen Patient:innen mit Depression die Mortalität in Abhängigkeit vom Schweregrad der Depression signifikant um das 1,8- bis 2,2-Fache erhöht ist [13].

Ein Screening auf das Vorliegen einer Depression ist insbesondere bei Patient:innen mit problematischer Diabeteseinstellung sinnvoll und zielführend. Hierfür hat sich der Zwei-Fragen-Test bewährt [28]:

1. Gab es in den letzten 4 Wochen eine Zeitspanne, während der Sie sich nahezu jeden Tag niedergeschlagen, traurig und hoffnungslos fühlten?
2. Gab es in den letzten 4 Wochen eine Zeitspanne, während der Sie das Interesse an Tätigkeiten verloren haben, die Ihnen sonst Freude machten?

Werden beide Fragen bejaht und wird ein durchgehender Zeitraum von mindestens zwei Wochen angegeben, sollte weiter untersucht werden, ob eine behandlungsbedürftige Depression vorliegt. Allerdings ist zu beachten, dass zwar die Sensitivität dieses Screening-Tests hoch, jedoch die Spezifität bei positivem Ergebnis niedrig ist, sodass neben einer ausführlichen Anamnese eine weiterführende Diagnostik z. B. mittels Patienten Gesundheitsfragebogen PHQ‑9 (Patient Health Questionnaire-9), Beck Depression Inventory, Hospital Anxiety and Depression Scale (HADS-D) oder WHO-Five-Well-Being Index (World Health Organization-5, http://who-5.org/) erforderlich ist [29–31]. Sollte nicht primär eine diabetesbezogene Maßnahme wie Diabetesschulung sinnvoll erscheinen, ist bei aufrechtem Verdacht auf eine psychische Erkrankung ab diesem Zeitpunkt jedenfalls eine Vorstellung bei einer/m Psychiater:in und Psychotherapeut:in in Erwägung zu ziehen – vor allem auch zur Abklärung einer akuten Selbstgefährdung.

Therapeutisch sollten Maßnahmen ergriffen werden, die sowohl die depressiven Symptome als auch die metabolische Situation aufgrund des Diabetes mellitus günstig beeinflussen. Bei den zielführenden Interventionen können unterschieden werden: 1. klinisch-psychologische und/oder psychotherapeutische Interventionen (z. B. Psychoedukation – s. auch Kapitel Schulung innerhalb dieses Positionspapieres, kognitiv-behaviorale Therapie), 2. (psycho)pharmakologische Behandlung und 3. Methoden zur Modifikation des Lebensstils.

### 1.3.1 Klinisch-psychologische und psychotherapeutische Interventionen

Psychodiabetologische Beratungs- und Behandlungsangebote sollen fixer Bestandteil der routinemäßigen Diabetesversorgung sein. Die frühzeitzeitige und niederschwellige klinisch-psychologische Unterstützung für alle Menschen mit Diabetes ist ein wesentlicher Beitrag, die Prognose des Diabetes zu verbessern und das Risiko für körperliche und psychische Folgeerkrankungen sowie neurokognitive Defizite zu minimieren. Im Falle von Therapieeskalation und Akutkomplikationen kann dem Risiko von Chronifizierung dysfunktionaler Verhaltensweisen im Rahmen des Diabetesselbstmanagements vorgebeugt werden.

Diabeteszentren und Diabetesambulanzen kommt bei der Identifikation von Personen mit erhöhtem Diabetes distress und subklinischen oder klinischen psychischen Störungen eine besondere Bedeutung zu. Die multidimensionale und interdisziplinäre Betreuung der Patient:innen erfordert dabei grundlegende Kenntnisse des gesamten Diabetesteams über diabetesbezogene psychische Belastungen und Komorbiditäten, um eine adäquate Versorgung zu gewährleisten.

Bereits im Rahmen von Diabetesschulungen sollen psychologische und psychosoziale Aspekte und die emotionale Ebene der Diabeteserkrankung thematisiert werden, um bereits geringe Ausprägungen von Diabetes distress und krankheitsbezogenen Belastungen aufzugreifen, zu reduzieren und die Lebensqualität von Menschen zu verbessern.

Zur Bedeutung von psychotherapeutischen Interventionen ist ein systematisches Review über 13 klinische Studien zur Behandlung von komorbid vorliegender Depression und Diabetes zu nennen [32]. Die angewendeten Interventionen waren höchst unterschiedlich in Bezug auf Zeitausmaß, Art der Anwendung (z. B. persönlich, telefonisch, internetbasiert), Inhalt (z. B. kognitiv behaviorale Therapie, Psychoedukation), intervenierende Berufsgruppe (z. B. Ärzt:innen, Pflegekraft, Psychotherapeut:innen). Kognitiv behaviorale Therapie (CBT) zeigte die höchste Evidenz, in mehreren Studien konnten moderate bis große antidepressive Effekte nachgewiesen werden. Diese Effektivität lässt sich noch steigern, wenn CBT mit weiteren psychologischen Techniken kombiniert wird, wie z. B. Stress-Management-Strategien, Coping-Skills-Training, Case-Management u. a. Eine solche Vorgangsweise ist in Bezug auf die positive Beeinflussung von depressiven Symptomen der alleinigen Verordnung von Antidepressiva überlegen [32, 33].

Die Studienergebnisse bezüglich positiver Effekte von psychologischen Interventionen auf die glykämische Kontrolle sind sehr heterogen, es gibt keine klare Evidenz. In der Mehrzahl der Untersuchungen ließen sich jedoch zumindest geringe positive Effekte nachweisen [32, 33].

### 1.3.2 (Psycho)pharmakologische Behandlung

Bei mittelgradigen und schweren depressiven Episoden wird die Verordnung von antidepressiver Medikation empfohlen und eine kontinuierliche psychiatrische Betreuung notwendig. Es ist zu beachten, dass das Nebenwirkungsprofil von antidepressiven Medikamenten je nach Substanzklasse sowie auch innerhalb derselben verschieden ist und eine an das Patient:innenprofil angepasste Auswahl des Therapeutikums erfordert [34, 35]. Häufige, für Patient:innen mit einer chronischen Stoffwechselerkrankung besonders relevante Nebenwirkungen umfassen gastrointestinale Symptome wie Übelkeit, Erbrechen und Diarrhö, weiters Sedierung, Agitation, Schlafstörung, Gewichtszunahme, Erhöhungen der Blutzuckerwerte, Verlängerung der QT-Zeit (faktisch alle SSRI[selektive Serotonin-Wiederaufnahmehemmer] und SNRI[Serotonin-Noradrenalin-Wiederaufnahmehemmer]), Hyperprolaktinämie, Hyponatriämie, Sexualfunktionsstörungen, Thrombozytenaggregationshemmung und andere [34, 35].

In rezenten systematischen Reviews und Metaanalysen lässt sich, wie für die allgemeine Bevölkerung, die Effektivität aller häufig verordneten Antidepressiva belegen [33]. Für SSRI ergibt sich eine moderate Effektivität im Vergleich zu Placebo sowie eine günstige Beeinflussung des HbA_1C_, wobei Letztere nicht generalisierbar erscheint [34–36].

Antidepressiva können über verschiedene Mechanismen zu Gewichtszunahme führen, wobei orexigene Effekte durch die Bindung an den Histamin-1-Rezeptor besonders relevant sind. Neben der Art des Antidepressivums scheint auch die Dauer der Anwendung das Ausmaß der Gewichtsveränderung zu beeinflussen. Die Antidepressiva Paroxetin, Mirtazapin sowie Trizyklika können die glykämische Kontrolle durch Steigerung der Insulinresistenz verschlechtern. SNRI sind gegenüber SSRI wegen des geringeren Risikos der Nebenwirkungen sexuelle Dysfunktion und Gewichtszunahme möglicherweise die zu bevorzugenden Substanzen. Eine Studie mit dem selektiven SNRI Milnacipran zeigte gute Effekte sowohl auf die depressive Symptomatik als auch sekundär auf Stoffwechselparameter ohne Auswirkungen auf die Sexualfunktion [15]. Agomelatin weist möglicherweise ein besonders günstiges Nebenwirkungsprofil mit Gewichtsneutralität auf [37]. Bupropion als Dopamin- und Noradrenalin-Wiederaufnahmehemmer ist mit Gewichtsabnahme assoziiert und hat keine Nebenwirkungen auf die Sexualfunktion, jedoch ein gering erhöhtes, dosisabhängiges Risiko für zerebrale Krampfanfälle [34–37].

Auswirkungen von Antidepressiva auf den Stoffwechsel sind in Tab. 1 dargestellt.

**Tab. 1:** Stoffwechselwirkungen von Antidepressiva. (Mod. nach Ress et al. [38]). (From: Psychische und neurokognitive Erkrankungen und Diabetes mellitus (Update 2023). Mental disorders and diabetes mellitus (Update 2023))

	Substanz	Gewicht	Plasmaglukose
Trizyklische Antidepressiva	Amitriptylin, Nortriptylin	±	±
MAO-Inhibitoren	Phenelzin, Tranylcypromin	+ + +	−
	Moclobemid	−	NA
SSRI	Citalopram, Paroxetin, Fluoxetin, Sertralin, u. a.	±	0/−
SNRI	Duloxetin, Venlafaxin, Milnacipran	+	0
Andere	Bupropion	−	0
	Mirtazapin	+ + +	0/+

Gewicht: + + + deutliche, + + moderate, + minimale bis keine Gewichtszunahme, − minimale bis keine Gewichtsabnahme, ± uneinheitliche Angaben

Plasmaglukose: 0/+ nicht eindeutig nachgewiesener Anstieg, 0/− nicht eindeutig nachgewiesene Reduktion, − Reduktion, ± unterschiedliche Angaben zu Auswirkungen auf die Plasmaglukose, 0 keine Auswirkungen

*NA* „no data available“

Der Einsatz antidiabetischer Medikation gegen depressive Symptome bei insulinresistenten Patient:innen fußt auf dem theoretischen Wissen, dass kognitive und affektive Störungen durch zentrale Insulinresistenz gefördert werden können [34, 35]. Die Substanz Pioglitazon, die Insulinresistenz reduziert, hat in 2 randomisierten kontrollierten Studien antidepressive Effekte gezeigt. Zusätzlich werden intranasales Insulin und inkretinbasierte injizierbare Therapien in Bezug auf mögliche antidepressive Effekte untersucht [39, 40].

Inkretinbasierte Diabetestherapien scheinen neben ihrer metabolischen Wirkung auch einen positiven Einfluss auf depressive Symptome, Angst, Suchtverhalten und emotionale Regulation zu haben. Über die Aktivierung zentraler GLP-1(„glucagon-like peptide-1“)- und GIP(„glucose-dependent insulinotropic peptide“)-Rezeptoren werden u. a. limbische und präfrontale Netzwerke moduliert, die für Affektregulation, Belohnungsverarbeitung und Impulskontrolle relevant sind.

Eine systematische Übersichtsarbeit und Metaanalyse mit über 107.000 Patient:innen zeigte, dass eine Behandlung mit GLP-1-Rezeptoragonisten im Vergleich zu Placebo nicht mit einem erhöhten Risiko für psychiatrische Nebenwirkungen oder einer Verschlechterung depressiver Symptome assoziiert war. Es kam zu Verbesserungen sowohl der körperlichen als auch der psychischen gesundheitsbezogenen Lebensqualität sowie zur Reduktion emotionalen Essverhaltens und zu einer Steigerung der Esskontrolle [41].

### 1.3.3 Lebensstilmodifikation

Der Versuch einer positiven Beeinflussung des Lebensstils der Betroffenen ist entscheidend, sowohl für die Besserung einer Depression als auch des Diabetes mellitus. Körperliche Aktivität, Schlafhygiene sowie Alkohol- und Tabakabstinenz verbessern die psychische und körperliche Gesundheit. Ebenso wirkt gesunde Ernährung protektiv sowohl für psychische als auch metabolische Erkrankungen.

## 1.4 Angststörungen

Angststörungen werden nach ICD-10-Klassifikation in phobische Störungen (Agoraphobie mit und ohne Panikstörung, soziale Phobie, spezifische [isolierte] Phobien), Panikstörung, generalisierte Angststörung, Angst und depressive Stimmung gemischt, Zwangsstörung sowie Reaktionen auf schwere Belastungen (akute Belastungsreaktion, posttraumatische Belastungsstörung) und Anpassungsstörungen eingeteilt. Auf Neuentwicklungen im ICD-11 wurde hingewiesen.

Angststörungen treten bei Patient:innen mit Diabetes häufiger auf als in der nichtdiabetischen Population, wobei lediglich für generalisierte Angststörungen und für Panikstörungen ausreichende Daten vorliegen [42, 43]. Speziell diabetesbezogene Ängste wie verstärkte und übermäßige Angst vor Hypoglykämien und vor diabetesspezifischen Folgeerkrankungen, müssen in der Anamnese berücksichtigt werden, da sie zu ungünstigen Auswirkungen auf die metabolische Kontrolle führen können.

Die Diagnostik der Angststörungen ist komplex und erfordert die Anwendung strukturierter klinischer Interviews, psychometrischer Fragebögen (z. B. Gesundheitsfragebogen zur Selbstbeurteilung und Schweregradmessung von Angststörungen = Generalized Anxiety Disorder 7 [GAD-7]) und diabetesspezifischer Fragebögen (z. B. Fragebogen zur Wahrnehmung von Unterzuckerungen) [44]. Für die Therapie ist ein Gesamtbehandlungsplan zu entwerfen, der in der Regel klinisch-psychologische Behandlung, Psychotherapie und medikamentöse Ansätze umfasst. Als Medikamente kommen je nach Erkrankung und eventueller Komorbidität in erster Linie Antidepressiva, aber auch Pregabalin und adjuvant Benzodiazepine zum Einsatz. Bei Letzteren ist die Gefahr der Abhängigkeitsentwicklung zu beachten, eine Dauerverordnung ist nicht indiziert.

## 1.5 Essstörungen

Die Klassifikation von Essstörungen erfolgt nach dem ICD-10-Code in Anorexia nervosa, Bulimia nervosa, Essattacken bei anderen psychischen Störungen, nicht näher bezeichnete Essstörungen und andere. Auf Neuentwicklungen im ICD-11 wurde hingewiesen.

Die häufig diskutierte Annahme der erhöhten Prävalenz von Anorexie und Bulimie konnte bei Typ-1-Diabetes bis dato in Studien nicht bestätigt werden. Allerdings kommt gestörtes Essverhalten, ohne die Diagnosekriterien für eine Essstörung zu erfüllen, in Kombination mit Typ-1-Diabetes häufiger vor als in der nichtdiabetischen Population [44, 45].

Für Patient:innen mit Typ-2-Diabetes liegen in der Literatur Hinweise vor, die auf ein vermehrtes Auftreten von Bulimie, Binge-Eating-Disorder und andere Essstörungen wie Night-Eating-Disorder schließen lassen. Allerdings ist die Datenlage inkonsistent [44, 45].

Zu beachten ist in jedem Fall, dass die Komorbidität Essstörung zu einer Verschlechterung der Stoffwechselsituation, zu einem vermehrten Auftreten von Retinopathie und Neuropathie und bei Menschen mit Diabetes mellitus Typ 1 zu einer erhöhten Frequenz von Krankenhausaufenthalten wegen diabetischer Ketoazidose führt [45].

Insbesondere bei jüngeren Patient:innen mit instabiler Metabolik und signifikanten Gewichtsschwankungen sollte an Bulimie, Binge-Eating-Disorder oder Insulin-Purging, das als Mittel zur Gewichtsreduktion eingesetzt wird, gedacht werden.

Die Diagnostik erfolgt mittels Anamnese sowie ergänzend durch strukturierte klinische Interviews und/oder Fragebögen bzw. gibt es für Menschen mit Diabetes mellitus adaptierte diagnostische Instrumente [46].

Die Therapie der Wahl bei Essstörungen besteht in psychotherapeutischen Interventionen, wie z. B. systemische Familientherapie, Verhaltenstherapie, Gesprächstherapie oder psychodynamische Therapie, sowie der klinisch-psychologischen Behandlung, eingebettet in einen Gesamtbehandlungsplan.

## 1.6 Aufmerksamkeitsdefizit‑/Hyperaktivitätsstörung (ADHS)

Bei diesem Krankheitsbild handelt es sich um die häufigste neurologische Entwicklungsstörung des Kindesalters. Sie ist charakterisiert durch entsprechend dem Entwicklungsstadium unangepasster und beeinträchtigender Unaufmerksamkeit, körperlicher Überaktivität, Impulsivität sowie häufig auch emotionaler Dysregulation. Seit etlichen Jahren ist ADHS mit einer Prävalenz von 8 % unter Kindern und Jugendlichen sowie 4–5 % unter Erwachsenen als häufige und lebenslange Erkrankung anerkannt. Männer weisen eine höhere Prävalenz als Frauen auf, wobei Frauen ein höheres Risiko von übersehenem ADHS haben.

Daten aus mehreren internationalen Studien zeigen deutliche Assoziationen zwischen den Risikofaktoren sowohl für ADHS als auch für Typ-2-Diabetes wie Übergewicht und Hypertonie. Insbesondere Übergewicht scheint dabei in einer bidirektionalen genetischen Beziehung zu ADHS zu stehen. Ebenso wurde ein vorbestehender maternaler Diabetes als Risikofaktor für ADHS unter Kindern festgestellt. Klinisch zeigten sich in mehreren Registerstudien höhere Raten an ADHS unter Menschen mit vorbestehendem Diabetes sowie an Diabetes bei bereits diagnostiziertem ADHS. Auch wenn die Ursachen für dieses gemeinsame Auftreten der beiden Diagnosen Gegenstand weiterer Forschungen sind, verdient diese Gruppe eine erhöhte Aufmerksamkeit. Verhaltensauffälligkeiten wie Unaufmerksamkeit und Desorganisation, wie sie mit ADHS assoziiert sind, erschweren bei komorbidem Diabetes mellitus möglicherweise das Selbstmanagement des Diabetes und die Therapieadhärenz [47, 48].

## 1.7 Emotional instabile Persönlichkeitsstörung

Bei der Komorbidität Diabetes mellitus und emotional instabile Persönlichkeitsstörung kommt es durch die z. T. erhebliche Beeinträchtigung der Impulskontrolle und Schwierigkeiten bei der Beziehungsgestaltung zu Auswirkungen auf die medizinische Betreuung. Die Patient:innen fallen z. B. durch besonders geringe Therapieadhärenz, selbstschädigendes Verhalten oder Substanzmissbrauch auf [49, 50]. Die altersstandardisierte Prävalenz von metabolischem Syndrom lag bei Patient:innen mit emotional instabiler Persönlichkeitsstörung 2‑fach höher als in der psychisch gesunden Kontrollgruppe (23,3 % vs. 10,6 %) [49]. Die Prävalenz von Diabetes mellitus wird mit 9 % angegeben [49]. Die Ursachen dafür dürften multifaktoriell sein, wobei auch teilweise die verordneten Psychopharmaka zur Diabetesmanifestation beitragen können.

Die wichtigste Basis für das weitere ärztliche Management besteht in der Herstellung einer tragfähigen Arzt-Patienten-Beziehung.

## 1.8 Schizophrene und bipolare Erkrankungen

Die Prävalenz von Typ-2-Diabetes ist bei Patient:innen mit schizophrenen, schizoaffektiven und bipolaren Erkrankungen 2‑ bis 3‑fach höher und auch die Manifestation von Typ-2-Diabetes im Durchschnitt 10 bis 20 Jahre früher als in der psychisch gesunden Bevölkerung. Die Ursachen dafür dürften nach aktuellem Wissensstand multifaktoriell sein, wobei folgende zu nennen sind: krankheitsspezifische Faktoren, niedriger sozioökonomischer Status, in dessen Folge ungesunder Lebensstil (Rauchen, ungesunde Ernährung, Bewegungsmangel) [4, 5, 51, 52], ungenügende medizinische Versorgung und nicht zuletzt ungünstige Nebeneffekte der meisten Antipsychotika [4, 5, 51]. Die meisten Studien zeigen das Auftreten von metabolischen Auffälligkeiten bald nach Behandlungsbeginn mit Neuroleptika. Bei ca. 12–13 % dieser Patient:innen führen diese metabolischen Veränderungen zu einem manifesten Diabetes mellitus. Ebenso kommt es zu einer Kumulation weiterer kardiovaskulärer Risikofaktoren wie Adipositas, Dyslipidämie und metabolischem Syndrom. In den Clinical Antipsychotic Trials of Intervention Effectiveness (CATIE) wurde gezeigt, dass ein Drittel der Patient:innen mit Schizophrenie signifikante unbehandelte metabolische und kardiovaskuläre Risikofaktoren aufwies [51].

Neuere wissenschaftliche Daten weisen auf einen gemeinsamen Vulnerabilitätsfaktor für Diabetes mellitus, metabolisches Syndrom und Schizophrenie hin. So konnten auch bei Patient:innen mit psychotischen Erstmanifestationen und vor Exposition gegenüber Psychopharmaka eine erhöhte Nüchternglukose sowie leicht erhöhte Raten an Insulinresistenz festgestellt werden. Insbesondere wird eine aberrante Aktivierung des Monozyten/Makrophagen-Systems mit abnormer Bildung von Zytokinen und Adipokinen als ursächlich diskutiert [52–54].

In einer schwedischen Register-Studie wurde bei 1.736.281 Teilnehmern untersucht, wie viele von ihnen im Untersuchungszeitraum von maximal 19 Jahren (median 10,6 Jahre), eine Schizophrenie, eine schizoaffektive Psychose und Typ-1- oder Typ-2-Diabetes manifestierten [55]. Dabei zeigte sich, dass Personen mit Schizophrenie ein deutlich höheres Risiko für Typ-1-Diabetes aufwiesen als Individuen ohne Schizophrenie (HR 2,84). Für schizoaffektive Psychosen konnte diese Koinzidenz nicht bestätigt werden. Das Risiko, Typ-2-Diabetes zu entwickeln, war für beide genannten psychiatrischen Krankheitsbilder signifikant erhöht, für Menschen mit Schizophrenie HR (95 % CI): 13,98 (8,70–22,46), *p* < 0,0001 und für Menschen mit schizoaffektiver Psychose HR (95 % CI): 14,27 (7,36–27,70), *p* < 0,0001. Bei den vielfältigen pathophysiologischen Mechanismen, die für die häufige Koinzidenz von Diabetes mellitus und Schizophrenie sowie schizoaffektive Psychose verantwortlich sind, spielen sowohl genetische Faktoren als auch Umweltfaktoren eine Rolle. Es ist anzunehmen, dass, ausgehend von gemeinsamen GEN-Funktionen im Gehirn oder Pankreas, bestimmte Kaskaden und Mechanismen aktiviert werden, die für die Manifestation der jeweiligen Komorbidität verantwortlich sind [55].

Die größte Herausforderung bei schwer verlaufenden Erkrankungen und in akuten Krisen ist die mangelnde bis fehlende Krankheits- und Behandlungseinsicht der Betroffenen. Ziel ist immer die Herstellung einer tragfähigen Ärzt:in-Patient:innen-Beziehung, auf deren Basis alle weiteren diagnostischen und therapeutischen Maßnahmen aufgebaut und koordiniert werden können. Dazu gehören im Sinne eines Gesamtbehandlungsplans eine möglichst individuell abgestimmte psychopharmakologische Behandlung, eine somatisch-medizinische Diagnostik und Betreuung sowie verschiedene unterstützende sozio- und psychotherapeutische Maßnahmen wie auch klinisch-psychologische Interventionen.

## 1.9 Neurokognitive Störungen

Die Klassifikation der Demenz erfolgt nach dem ICD-10-Code in Demenz bei Alzheimer-Krankheit, vaskuläre Demenz, Demenz bei andernorts klassifizierten Erkrankungen, nicht näher bezeichnete Demenz und andere Demenzformen. Auf Neuentwicklungen im ICD-11 wurde hingewiesen.

Sowohl mit Typ-1-Diabetes als auch mit Typ-2-Diabetes werden strukturelle zerebrale Veränderungen mit klinischen Manifestationen wie kognitive Defizite und Demenz assoziiert.

In großen, epidemiologischen Studien wurde gezeigt, dass vaskuläre und Alzheimer-Demenz bei Patient:innen mit Diabetes gehäuft auftreten. Bei Typ-2-Diabetes ist das Risiko für eine vaskuläre Demenz 2‑ bis 4‑fach erhöht und das Risiko, an einer Alzheimer-Demenz zu erkranken, 1,5- bis 2‑fach erhöht [56]. Der kausale Zusammenhang mit Diabetes mellitus ist wahrscheinlich durch das Auftreten von multiplen Risikofaktoren wie Glykämie, Hypertonie, Dyslipidämie, kardiale Erkrankungen u. a. zu erklären. Persistierende Hyperglykämie scheint eine wichtige Rolle bei der Entwicklung einer zerebralen Dysfunktion zu spielen [56, 57]. Passagere kognitive Einschränkungen durch akute Blutzuckerveränderungen wie Hypo- und Hyperglykämie sind bekannt und werden in diesem Kapitel nicht näher bearbeitet.

Die Heterogenität sowohl der diagnostischen Methoden als auch der untersuchten Populationen erschwert konklusive Aussagen über die Assoziation zwischen Typ-1-Diabetes und der Entwicklung von kognitiven Störungen. In der prospektiven Längsschnittstudie Epidemiology of Diabetes Interventions and Complications (EDIC-Nachfolgestudie des DCCT) über einen Zeitraum von 18 Jahren zeigten sich keine substanziellen kognitiven Defizite [58]. In einer Studie zu morphologischen Veränderungen des Gehirns wurden 95 jugendliche Patient:innen mit Typ-1-Diabetes und 49 Geschwister als Kontrollpersonen im Alter zwischen 7 und 17 Jahren untersucht. Die Frequenz von schweren Hypoglykämien und HbA_1c_-Werte als Ausdruck der Glykämieexposition wurden dokumentiert, und eine entsprechende Stratifizierung wurde vorgenommen. Patient:innen mit höherer Frequenz von schweren Hypoglykämien wiesen ein größeres Hippocampus-Volumen auf als Patient:innen mit einer niedrigeren Frequenz von schweren Hypoglykämien [59].

In einer Substudie der ACCORD-Studie, der ACCORD Memory in Diabetes(MIND)-Study, wurde der Zusammenhang zwischen Glykämie und kognitiver Funktion an einer großen Population mit Typ-2-Diabetes untersucht [60]. Der Glykämiestatus wurde mittels Nüchternblutzucker und HbA_1c_ ermittelt. Für die Beurteilung der kognitiven Funktion wurde der DSST(Digit Symbol Substitution-Test)-Score und für die Evaluierung der Hirnstruktur wurde eine Magnetresonanztomographie durchgeführt. Obwohl das totale Hirnvolumen in der Gruppe mit intensivierter Diabetestherapie signifikant höher war, zeigte sich kein Unterschied im DSST-Score zwischen intensivierter und Standardtherapie.

Für die Praxis relevant ist die ungünstige Auswirkung der kognitiven Defizite auf das erforderliche Selbstmanagement bei Diabeteserkrankungen. Für die Diagnostik eignen sich unter anderem folgende etablierte Tests: Demenz-Detektions-Test (DemTect), Mini-Mental-Status-Test (MMST) und Uhrentest.

## 1.10 Psychopharmaka und Stoffwechsel

Für die erhöhte Morbidität und Mortalität von Menschen mit psychischen Erkrankungen werden auch die teils stark ausgeprägten metabolischen Nebenwirkungen zahlreicher Psychopharmaka (viele Antipsychotika, manche Antidepressiva, Phasenprophylaktika) verantwortlich gemacht ([3–5, 51]; Tab. 2). Zu den wichtigsten metabolischen Nebenwirkungen zählen Gewichtszunahme, eine Erhöhung des Risikos, an Diabetes zu erkranken, und die Entwicklung eines atherogenen Lipidprofils.

**Tab. 2:** Stoffwechselwirkungen von Antipsychotika. (Mod. nach [34, 35, 41]). (From: Psychische und neurokognitive Erkrankungen und Diabetes mellitus (Update 2023). Mental disorders and diabetes mellitus (Update 2023))

Substanz	Gewichtszunahme	DM-Risiko	Dyslipidämierisiko
Clozapin	+ + +	+	+
Olanzapin	+ + + (5,0 kg)	+	+
Risperidon	+ + (2,0 kg)	DI	DI
Quetiapin	+ +	DI	DI
Aripiprazol	0/+	−	−
Ziprasidon	0/+ (0,6 kg)	−	−
Cariprazin	0/+	−	−

Zahlen in Klammern: Gewichtszunahme in Kilogramm

*DI* Datenlage inkonklusiv

+ minimal, + + moderat, + + + stark steigernder Effekt, 0/+ möglicher gering steigernder Effekt, − kein Effekt

Generell liegt ausreichende Evidenz für den Zusammenhang zwischen antipsychotischen Medikamenten und erhöhtem Risiko für Diabetes mellitus vor, allerdings ist dieses Risiko im Vergleich zu den traditionellen Diabetesrisikofaktoren klein. In einer Metaanalyse wurde das Diabetesrisiko durch die atypischen im Vergleich zu den konventionellen Antipsychotika mit 1,32 (95 % CI 1,15–15,1) angegeben [3, 4]. Die Evidenz für unterschiedliche Effekte der verschiedenen atypischen Antipsychotika ist weniger konklusiv, wobei das Ausmaß der Gewichtszunahme eine Rolle spielen dürfte. In der aktuellen Literatur finden sich nur wenige prospektive Studien zu diesem Thema. In einem systematischen Review der vorhandenen prospektiven randomisierten Studien zu der Frage: „Blutzucker und Schizophrenie“, finden sich keine konsistenten signifikanten Unterschiede [61]. Die kritische Frage: „Welcher Anteil des Risikos für die Manifestation metabolischer Störungen ist den Medikamenten zuzuordnen?“, kann bis dato nicht zufriedenstellend beantwortet werden. Die einseitige Assoziation zwischen kurzfristiger Gewichtszunahme unter Therapie mit atypischen Antipsychotika und dem Diabetesrisiko ignoriert alle anderen Ursachen von Gewichtszunahme bei Patient:innen mit schizophrenen Erkrankungen wie Umgebungsfaktoren oder spezielle Verhaltensmuster und Genetik (s. oben). In den meisten Studien sind häufige Komorbiditäten von schizophrenen Störungen wie Alkohol- oder Drogenmissbrauch bzw. -abhängigkeit und Depression nicht in die Überlegungen einbezogen [62].

Die Mechanismen, durch welche Antipsychotika Gewichtszunahme induzieren, sind tatsächlich weitgehend ungeklärt. Zahlreiche Hypothesen beschäftigen sich mit Effekten auf den Hypothalamus, einem Antihistamineffekt, einem sedierenden Effekt der Psychopharmaka mit Tagesmüdigkeit und daraus resultierender verminderter körperlicher Bewegung, einem Effekt auf die Leptinkonzentration und andere [4, 63].

Auch manche sogenannte Phasenprophylaktika, wie z. B. Lithiumsalze, Carbamazepin und Valproinsäure, können Auswirkungen auf den Stoffwechsel in Form von Gewichtszunahme und Hyperglykämie haben. Sie werden einerseits zur Akutbehandlung und Rückfallprophylaxe bei bestimmten affektiven Erkrankungen (bipolare Störung, unipolare Depression) oder schizoaffektiven Erkrankungen eingesetzt, andererseits sind manche von ihnen auch als Antiepileptika im Einsatz.

Eine medikamentös bedingte ausgeprägte Gewichtszunahme kann den Therapieerfolg entscheidend beeinflussen. Es kann zu einer belastenden Stigmatisierung der Betroffenen auf sozialer Ebene kommen. Daneben erfordern metabolische Nebenwirkungen zusätzliche therapeutische Maßnahmen, was in Summe zu Frustration, schlechter Therapieadhärenz oder Therapieabbruch führen kann. Abschließend ist noch zu erwähnen, dass sowohl typische als auch atypische Antipsychotika eine hohe Potenz für die Entwicklung einer Hyperprolaktinämie haben [3, 4].

## 1.11 Compliance – Adhärenz – Konkordanz

Bei der Behandlung von Menschen mit Diabetes mellitus wurde bereits vor geraumer Zeit festgestellt, dass das Konzept der „Compliance“ (Befolgung, Fügsamkeit) nicht geeignet ist bzw. nicht funktioniert [64]. Fünfundneunzig Prozent der Zeit ihres Lebens behandeln diese Patient:innen ihre Krankheit selbst und entwickeln daher ihre ganz persönlichen Strategien des Umgangs. Besser geeignet ist daher das Prinzip der „Adhärenz“. Patient:innen sollen für ein sinnvolles Vorgehen und Verhalten gewonnen werden [65]. Das in der Sozialpsychiatrie bewährte Modell des Empowerments ist auch in der Behandlung von Menschen mit Diabetes sehr geeignet [66]. Ziel ist es, die Patient:innen in die Lage zu versetzen bzw. dabei zu unterstützen, dass sie selbst ihr Leben und auch ihre gesundheitlichen Probleme meistern können. Nur wenn es gelingt, eine „gemeinsame Landkarte“ zu entwerfen, wenn die Ziele von Arzt und Patient:innen übereinstimmen, wird die Zusammenarbeit gut funktionieren und in der Folge die Diabeteseinstellung zufriedenstellend sein. In diesem Sinne wird in der Literatur auch der Begriff „Concordance“ verwendet [67].

## 1.12 Diabetes und Selbstgefährdung

Generell ist bei allen Patient:innen mit Diabetes mellitus und psychischer Komorbidität mit einer potenziellen chronischen Selbstgefährdung zu rechnen, v. a. durch mangelnde Therapieadhärenz. Ebenso ist eine erhöhte Suizidgefährdung anzunehmen, es gibt jedoch keinen Konsens bezüglich der Höhe des Risikos, z. B. stellte eine britische Studie fest, dass Patient:innen mit Typ-1-Diabetes eine 11-fach erhöhte Rate von Suizidversuchen mit Insulin aufweisen [68]. Eine Untersuchung von Anfragen an eine Vergiftungszentrale ergab, dass ca. 0,16 % aller Anfragen Überdosierungen mit Insulin betrafen, davon 90 % in suizidaler oder parasuizidaler Absicht [69]. Immerhin beträgt die Letalität der Verabreichung von sehr hohen Insulindosen in suizidaler Absicht 3–10 %, als Dauerschaden kann es durch schwere Unterzuckerung zu einer Enzephalopathie kommen. Depressive Patient:innen mit Diabetes mellitus und Insulintherapie müssen daher ärztlich besonders sorgfältig geführt werden. Dazu gehören nicht nur eine genaue Anamnese mit Erfassung von Risikofaktoren, sondern auch eine psychiatrische Mitbehandlung und Psychotherapie sowie bei Bedarf Unterstützung durch andere Personen z. B. zur Insulinverabreichung.

## 1.13 Diabetesschulungen, klinisch-psychologische und psychotherapeutische Begleitung

Sorgfältiges Selbstmanagement ist bei Patient:innen mit Diabetes mellitus besonders wichtig, um Akutkomplikationen wie Hyper- und Hypoglykämie und Langzeitschäden wie Mikro- und Makroangiopathie zu vermeiden. Der Erwerb von Kenntnissen über Ursachen, Verlauf und Therapie der Krankheit und von bestimmten Fertigkeiten ist Voraussetzung für die effektive und dauerhafte Umsetzung von therapeutischen Maßnahmen im persönlichen Alltag. Eine Diabetesschulung, die die besonderen Gegebenheiten bei psychisch kranken Patient:innen berücksichtigt, ist daher ein wesentlicher Grundstein für die Behandlung. Zu den besonderen Problemen bei dieser Patientengruppe zählen Gewichtszunahme durch bestimmte Psychopharmaka, erhebliche Tagesmüdigkeit, unstrukturierter Tagesablauf sowie wenig Krankheitseinsicht, die die Umsetzung gesundheitsfördernder Maßnahmen behindern können.

In der rezenten Literatur finden sich wenige Daten zum Thema Diabetesschulung bei Patient:innen mit psychischer Komorbidität. Eine ältere Untersuchung zeigt, dass die Kombination aus Diabetesschulung und kognitiver Verhaltenstherapie bei Patient:innen mit Typ-2-Diabetes und Depression signifikant bessere Auswirkungen auf den HbA_1c_-Wert zeigte als Diabetesschulung allein [70]. In einer anderen Studie konnte bei diabetischen Patient:innen mit Depression ein ähnlicher integrativer Ansatz bestätigt werden [71].

Obwohl die positiven Auswirkungen von kognitiver Verhaltenstherapie und anderen Psychotherapieformen auf depressive Symptome außer Frage stehen, sind Auswirkungen von psychotherapeutischer Begleitung allein (ohne strukturierte Diabetesschulung) auf Stoffwechselparameter weniger gut belegt. Studien zu dieser Fragestellung weisen in der Regel kleine Fallzahlen und kurze Dauer auf [72–74]. In einem systematischen Review bestätigte sich, dass nichtpharmakologische Therapien wie psychologische Behandlung und Psychotherapie zwar die depressiven Symptome reduzierten, jedoch häufig nicht zu einer Verbesserung der Metabolik führten [75].

Ein ideales Modell für psychisch kranke Menschen mit Diabetes würde aus einer Kombination aus strukturierter Diabetesschulung (mit Adhärenztraining), klinisch psychologischen Interventionen und Psychotherapie bestehen, wobei durch diesen integrativen Ansatz sowohl eine Besserung der Depressionssymptome als auch ein besseres Verständnis von Diabetes mellitus mit Steigerung der Therapieadhärenz erreicht werden könnten.

## 1.14 Empfehlungen

Um bei Patient:innen mit der Besonderheit somatische und psychische Komorbidität das metabolische und kardiovaskuläre Risiko zu reduzieren, sollten sowohl definierte Screening- und Monitormaßnahmen wahrgenommen werden [1, 2, 5, 8, 9, 45, 76, 77], als auch bestimmte therapeutische Schritte umgesetzt werden:

1. Die Erhebung des psychosozialen Status sollte als essenzieller Bestandteil in die Betreuung diabetischer Patient:innen eingeführt werden. Dieser umfasst Lebenssituation und Lebensqualität, Stimmung, Einstellung zur Erkrankung, Erwartungen im Hinblick auf Krankheitsverlauf und Therapie, Verfügbarkeit von Ressourcen und andere.
2. Darüber hinaus sollten alle Patient:innen mit Diabetes mellitus einmal jährlich auf das Vorliegen folgender psychischer Erkrankungen gescreent werden: diabetesspezifischer Stress („diabetes distress“), Depression, Angststörung und Essstörung. Vor allem in bestimmten kritischen Phasen im Krankheitsverlauf wie Erstmanifestation, dem Auftreten von Akut- oder Langzeitkomplikationen oder bei relevanten Therapieumstellungen soll ein Screening erfolgen. Ein jährliches Screening auf kognitive Dysfunktion und Demenz empfiehlt sich bei Patient:innen über 65 Jahre.
3. Bei psychisch kranken Patient:innen mit antipsychotischer oder antidepressiver Therapie ist die Erhebung kardiovaskulärer und metabolischer Risikofaktoren zu Beginn der Therapie und im Verlauf sinnvoll [1, 2, 5, 21, 76–78]. Folgende Screening- und Monitoring-Empfehlung hat sich in der Praxis bewährt: zumindest einmal jährliches Screening im Hinblick auf die Risikofaktoren Diabetes mellitus, Dyslipidämie, viszerale Adipositas und Hypertonie. Messungen von Blutdruck, Bauchumfang und Gewicht sollten einen Monat nach Beginn der Therapie mit Psychopharmaka und anschließend in Abständen von 3 bis 6 Monaten durchgeführt werden. Die Messung des HbA_1c_ ist in 3‑monatigen Abständen sinnvoll. Bestimmungen von Prolaktin im Serum sind bei für Hyperprolaktinämie prädisponierender Psychopharmakatherapie ein- bis zweimal jährlich empfehlenswert ([34, 35]; Tab. 3). Schulungen für diese spezielle Patientengruppe sollten angeboten werden. Die prophylaktische Etablierung einer niedrig dosierten Metformin-Therapie ist bei ungünstiger Entwicklung in Richtung metabolisches Syndrom unter Psychopharmakatherapie in Erwägung zu ziehen, bedeutet jedoch einen „Off-Label“-Einsatz.
4. Diese Screening- und Monitorleitlinie sowie therapeutische Interventionen wie Umstellung der Ernährung, regelmäßige körperliche Bewegung, Motivation zu Tabak- und Alkoholkarenz sollen in interdisziplinärer Kooperation zwischen Allgemeinmedizinern, Internisten, Endokrinologen und Psychiatern umgesetzt werden.
5. Die antihyperglykämische Therapie sollte prinzipiell auch bei Menschen mit Typ-2-Diabetes und komorbider psychischer oder neurokognitiver Erkrankung entsprechend den Leitlinien der ÖDG umgesetzt werden (s. Kapitel Antihyperglykämische Therapie bei Typ-2-Diabetes). Insgesamt ist die Studienlage zu antihyperglykämischer Therapie in dieser Patientengruppe spärlich. Wie bereits oben beschrieben, kann bei viszeraler Adipositas und anderen Parametern des metabolischen Syndroms unter antipsychotischer Therapie der frühe Einsatz von Metformin (vor Diabetesmanifestation) in Erwägung gezogen werden.
  Die sehr gute Wirkung von GLP-1-Rezeptoragonisten auf Blutzucker, Gewicht und Lipide bei Menschen mit Typ-2-Diabetes und psychischer Komorbidität wird in mehreren Studien bestätigt [78–80]. Eine Analyse zeigte, dass die Behandlung mit GLP-1-Rezeptoragonisten bei antipsychotisch therapierten Patient:innen mit Schizophrenie im Vergleich zur Kontrollgruppe sicher und wirksam in Bezug auf kardiometabolische Parameter ist [81].
  Hinsichtlich weiterer positiver Wirkungen auf das Gehirn und die Psyche gibt es vielversprechende Ergebnisse sowohl für GLP-1- als auch für duale GLP-1/GIP-Rezeptoragonisten. Fakt ist, dass bei Typ-2-Diabetes und Adipositas durch erhöhte Bildung reaktiver Sauerstoffspezies (ROS) und subklinische Inflammation die Entwicklung von zentraler Neuroinflammation und zerebralem oxidativem Stress gefördert wird. Beide gelten unter anderen als wesentliche Merkmale der Neuropathologie von Morbus Alzheimer [82]. Derzeit werden neuroprotektive und antiinflammatorische Wirkungen von GLP-1- und dualen GLP-1/GIP-Rezeptoragonisten, die potenziell bei neuropsychiatrischen Erkrankungen wie Morbus Alzheimer und Morbus Parkinson von Nutzen sein könnten, intensiv erforscht [40].
  Für SGLT-2-Hemmer liegen zur Anwendung bei psychisch kranken Menschen keine spezifischen Daten vor. Die Frage nach einer möglicherweise kumulativen Inzidenz von Ketoazidosen durch die Kombination von SGLT-2-Hemmern und Antipsychotika (beide Pharmaka weisen eine erhöhte Inzidenz für Ketoazidosen auf) kann derzeit nicht beantwortet werden [83]. DPP4-Hemmer und Glitazone sollen entsprechend den Leitlinien der ÖDG eingesetzt werden. Komplexe Insulintherapien können bei bestimmten psychischen Erkrankungen zur Überforderung und konsekutiv zu hyperglykämischen Entgleisungen führen.

**Tab. 3:** Monitoring von Risikofaktoren nach Initiierung einer psychopharmakologischen Therapie. (Mod. nach De Hert et al. [3, 5]). (From: Psychische und neurokognitive Erkrankungen und Diabetes mellitus (Update 2023). Mental disorders and diabetes mellitus (Update 2023))

	Therapiebeginn	Woche 4	Woche 12–26	Jährlich
Vorerkrankungen	X	–	–	–
Familienanamnese	X	–	–	–
Bewegung	X	–	–	X
Ernährung	X	–	–	X
Nikotin	X	–	–	X
Beratung RF	X	X	X	X
Gewicht	X	X	X	X
BU	X	X	X	X
Blutdruck	X	X	X	X
BZ nüchtern	X	X	X	X
HbA_1c_^a^	X	–	X^a^	X^a^
Lipide	X	X	X	X
Prolaktin	X	–	–	X

^a^In 3‑monatigen Abständen

## Interessenkonflikt

H. Abrahamian hat von folgenden Unternehmen, die auch fördernde Mitglieder der ÖDG sind, Forschungsunterstützungen und/oder Honorare erhalten: Amgen, Boehringer Ingelheim, Novo Nordisk, Sanofi-Aventis, Servier. Zusätzlich gibt H. Abrahamian an, dass für sie persönlich kein Interessenkonflikt vorliegt. A. Kautzky-Willer hat von folgenden Unternehmen, die auch fördernde Mitglieder der ÖDG sind, Forschungsunterstützungen und/oder Honorare erhalten: AstraZeneca, Bayer Health Care, Böhringer Ingelheim, Eli Lilly, Merck Sharp & Dohme, Novartis, Novo Nordisk, Roche, Amarin. A. Rießland-Seifert hat von folgenden Unternehmen, die auch fördernde Mitglieder der ÖDG sind, Forschungsunterstützungen und/oder Honorare erhalten: AstraZeneca, Bristol-Myers Squibb, Eli Lilly, Lundbeck, A. Menarini Österreich. A. Kautzky hat von folgenden Unternehmen Honorare erhalten: Takeda, Pfizer. J. Brix hat von folgenden Unternehmen, die auch fördernde Mitglieder der ÖDG sind, Forschungsunterstützungen und/oder Honorare erhalten: Abbott, AstraZeneca, Bayer Health Care, Böhringer Ingelheim, Dexcom, Eli Lilly, Medtronic, Novo Nordisk. P. Fasching hat von folgenden Unternehmen Forschungsunterstützungen und/oder Honorare erhalten: Bayer Health Care, Boehringer Ingelheim, Daiichi-Sankyo, Eli Lilly, Novo Nordisk. Zusätzlich gibt P. Fasching an, dass für ihn persönlich kein Interessenkonflikt vorliegt. Er legt offen, dass er in diesem Zeitraum von den genannten Firmen Honorare für Vorträge und Consulting erhalten hat bzw. Fortbildungsunterstützung im Rahmen der (dienst)rechtlichen Rahmenbedingungen bezogen hat (Einladung zu Kongressen) bzw. klinische Studien als PI mit einzelnen Firmen durchgeführt hat bzw. durchführt. H. Toplak hat von folgenden Unternehmen, die teilweise fördernde Mitglieder der ÖDG sind, Forschungsunterstützungen und/oder Honorare erhalten: Amgen, Boehringer Ingelheim, Bristol-Myers Squibb, Eli Lilly, Merck Sharp & Dohme, Novartis, Novo Nordisk, Pfizer, Sanofi-Aventis. B. Harb, D. Beer und D. Lebherz-Eichinger geben an, dass kein Interessenkonflikt besteht.
